# Behavioral and electrophysiological effects of endocannabinoid and dopaminergic systems on salient stimuli

**DOI:** 10.3389/fnbeh.2014.00183

**Published:** 2014-05-19

**Authors:** Daniela Laricchiuta, Alessandra Musella, Silvia Rossi, Diego Centonze

**Affiliations:** ^1^IRCCS Fondazione Santa LuciaRome, Italy; ^2^Dipartimento di Psicologia, Facoltà di Medicina e Psicologia, Università “Sapienza” di RomaRome, Italy; ^3^Dipartimento di Neuroscienze, Università Tor VergataRome, Italy

**Keywords:** conflicting tasks, approach/avoidance motivation, haloperidol, URB597, AM251

## Abstract

Rewarding effects have been related to enhanced dopamine (DA) release in corticolimbic and basal ganglia structures. The DAergic and endocannabinoid interaction in the responses to reward is described. This study investigated the link between endocannabinoid and DAergic transmission in the processes that are related to response to two types of reward, palatable food and novelty. Mice treated with drugs acting on endocannabinoid system (ECS) (URB597, AM251) or DAergic system (haloperidol) were submitted to approach-avoidance conflict tasks with palatable food or novelty. In the same mice, the cannabinoid type-1 (CB_1_)-mediated GABAergic transmission in medium spiny neurons of the dorsomedial striatum was analyzed. The endocannabinoid potentiation by URB597 magnified approach behavior for reward (food and novelty) and in parallel inhibited dorsostriatal GABAergic neurotransmission. The decreased activity of CB_1_ receptor by AM251 (alone or with URB597) or of DAergic D_2_ receptor by haloperidol had inhibitory effects toward the reward and did not permit the inhibition of dorsostriatal GABAergic transmission. When haloperidol was coadministered with URB597, a restoration effect on reward and reward-dependent motor activity was observed, only if the reward was the palatable food. In parallel, the coadministration led to restoring inhibition of CB_1_-mediated GABAergic transmission. Thus, in the presence of simultaneous ECS activation and inhibition of DAergic system the response to reward appears to be a stimulus-dependent manner.

## Introduction

Survival strictly depends on the drive with which subjects seek natural reinforcers, such as food, water, sex, and maternal care. Converging electrophysiological, biochemical, and behavioral evidence implicates the endocannabinoid system (ECS) in the neurobiological events that attribute value to various types of reinforcers (Maldonado et al., 2006; Marsicano and Lutz, 2006; Laricchiuta et al., 2012a, b). The ECS mediates the balance between approach and avoidance behaviors toward stimuli in humans (Van Laere et al., 2009) and rodents (Lafenêtre et al., 2009; Laricchiuta et al., 2012a,and the Open-Field test with b), the detailed mechanism of which is unknown.

Most central ECS functions are mediated by cannabinoid type-1 (CB_1_) receptors (Freund et al., 2003; Piomelli, 2003) that presynaptically inhibit primarily glutamatergic and GABAergic transmission, but also dopaminergic and cholinergic transmission (Matias and Di Marzo, 2007; Kano et al., 2009). Notably, the reward effects of primary reinforcers or environmental stimuli that are associated with food or drug intake have been related to enhanced dopamine (DA) release in corticolimbic and basal ganglia structures (Bassareo et al., 2002; Ito et al., 2002; Lupica and Riegel, 2005). Thus, DA is a neurosignal that has been evolutionarily adjusted to boost the organism toward reinforcer (Alcaro and Panksepp, 2011).

There is increasing evidence that implicates a dynamic DAergic and endocannabinoid interaction in neuronal, endocrine, and metabolic responses to reward (Di Marzo et al., 2004; Fernández-Ruiz et al., 2010). The inhibitory effects of the ECS against DAergic neurons in mesolimbic structures have been hypothesized to attribute the salience to reward. Supporting this model, CB_1_ antagonists impair the attribution of salience to a stimulus (De Vries et al., 2001; Fattore et al., 2003). Further, the ECS has been implicated in several neuropsychiatric conditions, including DA-related disorders, such as schizophrenia (Emrich et al., 1997), Parkinson disease (Maccarrone et al., 2003), and drug addiction (Maldonado and Rodríguez de Fonseca, 2002). In these pathological conditions, involvement of the ECS likely reflects the activity of reward circuitry, which comprises midbrain DAergic neurons and their target structures (Berke and Hyman, 2000; Everitt and Wolf, 2002).

This study aimed to behaviorally and electrophysiologically examine endocannabinoid and DAergic involvement in processes that are related to seeking two types of reward: palatable food and novelty. The interaction between the ECS and DAergic system in processing salient information is attracting significant interest.

In this study, mice that were treated with drugs that act on the ECS or DAergic system were subjected to approach-avoidance conflict tasks with two rewards (food or novelty). In addition, CB_1_-mediated GABAergic transmission in medium spiny neurons of the dorsomedial striatum was analyzed. This area is the ideal structure to study the interplay between the ECS or DAergic systems, because it has a high density of CB_1_ and DAergic receptors (Piomelli, 2003) and is critical in goal-directed behaviors (Koob and Volkow, 2010).

## Materials and methods

### Subjects

Young (32 ± 2 days) male C57BL/6JOlaHsd mice (Harlan, Italy) were used. All animals were housed, 4 per cage, with food (Mucedola 4RF21, Italy) and water *ad libitum*. The mice were kept under a 12-h light/dark cycle with the lights coming on at 07:00 h, controlled temperature (22–23°C), and constant humidity (60 ± 5%). All efforts were made to minimize animal suffering and reduce the number of animals that were used, per the European Directive (2010/63/EU).

All animals (*n* = 10/group) included in the present study were behaviorally tested in the Approach/Avoidance Y-Maze (A/A Y-Maze) and the Open-Field test with novel object. Both tasks permit to detect crucial components of animal behavior linked to approach-avoidance motivation, as the tendency to explore, respond to an environmental change or seek for reward and novelty. In fact, the explorative drive represents the prerequisite of the recognition and seeking for novel stimuli, crucial components of approach and avoidance motivation.

**Figure 1:**
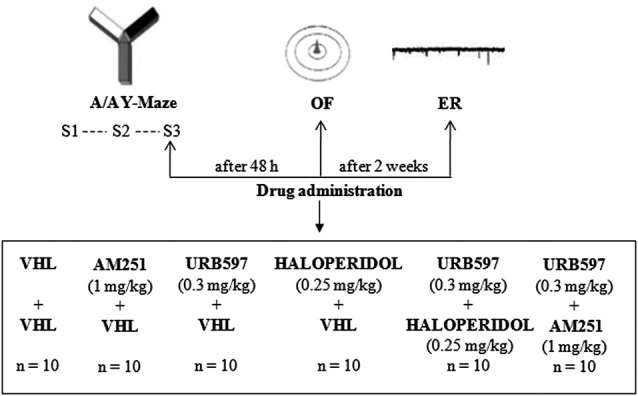
**Procedures and global timing of experimental design.** C57BL/6J mice were tested in the Approach/Avoidance Y-Maze (A/A Y-Maze) and in the Open Field (OF) test. At the end of behavioral testing, Electrophysiological Recordings (ER) were performed from medium spiny neurons of the dorsomedial striatum. Drugs were administered before S3 of A/A Y-Maze and OF task as well as before ER.

Experimental procedures are shown in Figure 1.

### Behavioral testing: Approach/Avoidance Y-Maze (A/A Y-Maze)

This test was performed as previously described (Laricchiuta et al., 2012a, b). In particular, the apparatus consisted of a Plexiglas Y-maze with a starting gray arm from which two arms (8 cm × 30 cm × 15 cm) stemmed, arranged 90° to each other. A T-guillotine door was placed at the end of the starting arm to prevent the animal from returning to the start. An arm entry was defined as four legs entering one of the arms. The two arms of choice differed in color and brightness—one of the two arms had a black and opaque floor and walls and no light inside, whereas the other had a white floor and walls and was lit by a 16-W neon lamp. Notably, the colored “furniture” and the neon lamp were exchangeable between arms to alternate the position of the white and black arms.

The apparatus was placed in a dim room that was lit by a red light (40 W) and was cleaned thoroughly with 70% ethanol and dried after each trial to remove scent cues. At the end of each arm of choice, there was a blue chemically inert tube cap (3 cm in diameter, 1 cm deep) that was used as a food tray. The depth of the tray prevented mice from seeing the reward at a distance but permitted easy reward—i.e., eating. Because the appetites for palatable foods must be learned (Lafenêtre et al., 2009), 1 week before the behavioral testing, the animals were exposed to a novel palatable food (Fonzies, KP Snack Foods, Munchen, Germany) in their home cages for three consecutive days (Bassareo et al., 2002). Fonzies (8% protein, 33% fat, and 53% carbohydrate, for a caloric value of 541 kcal/100 gm) consist of corn flour, hydrogenate vegetable fat, cheese powder, and salt.

At the start of behavioral testing, the mice were subjected to a 1-day habituation phase in which all Y-Maze arms were opened to encourage exploration of the maze without the presence of food. The white and lit arm was placed on the right side of the apparatus for the first 5 min and then on the left side for the subsequent 5 min. To increase the motivation to search for the reward, the animals were slightly food-deprived by limiting access to food in the 10 h before the test; this procedure did not result in any body weight loss.

The testing phase (24 h after the habituation phase) comprised two 10-trial sessions. In Session 1 (S1), the animal was placed in the starting arm and could choose to enter one of the two arms, both containing the same standard food reward. After eating, the animal was allowed to stand in its cage for a 1-min intertrial interval. At the end of each trial, the reward was replaced. The spatial position of each arm (black and dark or white and lit) was sequentially exchanged and side-balanced during the entire test to exclude any preference with regard to side.

During Session 2 (S2), starting 24 h after S1, the white arm was rewarded with the highly palatable food (Fonzies), and the black arm was rewarded with the standard food pellet.

Forty-eighty hours after S2, the animals were retested (S3) in the A/A Y-Maze per the S2 protocol.

The parameters were: *white choices*, the frequency of entries into the white arm, to study the approach and avoidance components; *entry* *latency* in the arm of choice, to study the locomotor component; and *defecation boluses*, to evaluate anxiety levels.

### Behavioral testing: Open-Field (OF) test with novel object

Forty-eighty hours after the end of the A/A Y-Maze task, the OF test was performed. The apparatus, placed in a dimly lit (red light 40 W) and soundproof cubicle room, consisted of a circular arena (diameter 60 cm) that was delimited by a pale gray wall that was 20 cm high. During Session 1 (S1), a single animal was allowed to explore the empty open field, and its baseline level of activity was measured. During Session 2 (S2), a new appealing object (a gray plastic cone: height: 10 cm, diameter: 6 cm, with a circular base: diameter: 9.5 cm) was inserted in the anxiogenic central location of the arena. Thus, the response to change exhibited by the animal in S2 is an index of its reaction to conflicting situation.

Sessions lasted 10 min, and the intersession interval was 5 min. The apparatus and object were cleaned thoroughly with 70% ethanol and dried after each session to remove scent cues.

The entire test was recorded on a video camera that was mounted on the ceiling. The video feed was relayed to a monitor and processed through an image analyzer (Ethovision, Noldus, Wageningen, the Netherlands).

The parameters that we considered were: *total distance* (in cm) traveled in the arena and mean *velocity*, to study the explorative and locomotor components; *peripheral distance* (expressed as the percentage of total distance traveled in a 6-cm peripheral annulus), *freezing time* and *defecation boluses*, to evaluate anxiety levels; and *contact* *times* with the object, to study the approach-avoidance components that were elicited by insertion of the new appealing object in an anxiogenic central location of the wide arena. Contact with the object was considered to take place when the mouse’s snout touched it, or when the animal sniffed the object for at least 1 s.

### Electrophysiological recordings

Mice were killed by cervical dislocation under halothane anesthesia. By using a vibratome, corticostriatal coronal slices (200 µm) were prepared from brain fresh tissue and left recovered for 30 min in an Artificial CSF (ACSF) gassed with 95% O_2_–5% CO_2_. The composition (in mM) of the control ACSF was: NaCl (126), KCl (2.5), MgCl_2_ (1.2), NaH_2_PO_4_ (1.2), CaCl_2_ (2.4), Glucose (11), NaHCO_3_ (25).

Whole-cell patch clamp recordings from single striatal neurons were made as previously described (Centonze et al., 2007a, b; De Chiara et al., 2010a, b; Laricchiuta et al., 2012b; Musella et al., 2014). A single slice was transferred to a recording chamber and submerged in a continuously flowing oxygenated ACSF (34°C, 2–3 ml/min). The striatum could be readily identified under low power magnification, whereas individual neurons were visualized *in situ* using a differential interference contrast (Nomarski) optical system. This system employed an Olympus BX50WI (Japan) non-inverted microscope with 40x water immersion objective combined with an infra-red filter, a monochrome CCD camera (COHU 4912), and a PC compatible system for analysis of images and contrast enhancement (WinVision 2000, Delta Sistemi, Italy). Recording pipettes were advanced towards individual striatal cells in the slice under positive pressure and visual control (WinVision 2000, Delta Sistemi, Italy) and, on contact, tight GΩ seals were made by applying negative pressure. The membrane patch was then ruptured by suction and membrane current and potential monitored by using an Axopatch 1D patch clamp amplifier (Molecular Devices, Foster City, CA, USA). Whole-cell access resistances measured in voltage clamp were in the range of 5–20 MΩ.

Whole-cell patch clamp recordings were made with borosilicate glass pipettes (1.8 mm o.d.; 2–3 MΩ), in voltage clamp mode, at the holding potential (HP) of −80 mV.

To detect spontaneous GABA_A_-mediated inhibitory postsynaptic currents (sIPSCs), the intraelectrode solution comprised (in mM): CsCl (110), K^+^-gluconate (30), ethylene glycol-bis (ß-aminoethyl ether)-N,N,N′,N′-tetra-acetic acid (EGTA; 1.1), HEPES (10), CaCl_2_ (0.1), Mg-ATP (4), and Na-GTP (0.3). MK-801 (30 µM) and CNQX (10 µM) were added to the external solution to block NMDA and non-NMDA glutamate receptors, respectively.

sIPSCs were stored using a P-CLAMP 9 (Molecular Devices, Foster City, CA, USA) and analyzed offline on a personal computer with Mini Analysis 5.1 (Synaptosoft, Leonia, NJ, USA). The offline analysis was performed on sIPSCs that were recorded during fixed times (5–10 samples of 2–3 min each, recorded every 2–3 min). Only cells that had stable frequencies (<20% change in the control samples) were considered.

HU210, used in the slices, was first dissolved in DMSO and then in ACSF to the desired final concentration. DMSO alone was used as the control. The concentrations (in µM) of the various drugs were as follows: CNQX (10), HU210 (1), and MK-801 (30) (Tocris, Bristol, UK).

For the electrophysiological data, “n” refers to the number of cells. One to six neurons per animal were recorded. Electrophysiological measures were obtained by pooling data from at least five animals in each group.

### Drugs

All drugs were dissolved in vehicle (VHL) that comprised saline solution with 10% DMSO and 5% Tween 80 and administered at volume of 5 ml/kg of body weight. The animals in the URB group (*n* = 10) were first intraperitoneally (i.p.) injected with the fatty acid amide hydrolase (FAAH) inhibitor URB597 (0.3 mg/kg; Alexis, USA) and then injected immediately after with the same volume of VHL without drug. The animals in the AM group (*n* = 10) were injected i.p. first with the CB_1_ receptor inverse agonist AM251 (1 mg/kg; Tocris, UK) and immediately after with the same volume of VHL without drug. In the haloperidol (HAL) group (*n* = 10), animals were injected i.p. first with the DAergic D_2_ receptor antagonist haloperidol (0.25 mg/kg; Sigma, Italy) and immediately after with the same volume of VHL without drug. The URB+HAL group (*n* = 10) was injected i.p. with URB597 (0.3 mg/kg) and haloperidol (0.25 mg/kg). The animals in the URB+AM group (*n* = 10) were injected i.p. with URB597 (0.3 mg/kg) and AM251 (1 mg/kg). The control animals (*n* = 10) received the same volume of VHL i.p. twice.

Further, we examined 10 animals that were injected i.p. with AM251 (1 mg/kg) and haloperidol (0.25 mg/kg), but it was not possible to report their results, because they were completely inhibited and remained motionless, failing to seek the reward in either behavioral test.

Drugs were administered at dosages which are reported to have effects on reward-, novelty- and emotion-related behaviors. Namely, the selection of URB597 dosage at 0.3 mg/kg was based on our previous behavioral and electrophysiological results (Rossi et al., 2010; Laricchiuta et al., 2012b, 2013). The same dosage was used by Naderi et al. (2008) to investigate URB597 behavioral effects on anxiety, as well as by Fegley et al. (2005) to characterize its neurochemical profile.

The selection of AM251 dosage at 1 mg/kg was based on behavioral results on locomotor- (Eisenstein et al., 2010), anxiety- (Umathe et al., 2009) and reward- (Xi et al., 2008) related effects. The same dosage was used by Maione et al. (2013) to investigate AM251 neurochemical properties.

The selection of haloperidol dosage at 0.25 mg/kg was based on behavioral results on locomotion and exploration (Karlsson et al., 2008; Chatterjee et al., 2011).

Based on their pharmacokinetic properties (Kathuria et al., 2003; Patel and Hillard, 2006), the drugs were administered 30 min before S3 of the A/A Y-Maze and S1of the OF task. Two weeks later, the animals were reinjected with the same drugs that they had received and sacrificed 30 min later to take electrophysiological recordings (ER) of the striatal activity. The experimenters that performed the behavioral testing and ER were blind to the drug treatment.

### Statistical analysis

Data were presented as mean ± SEM and tested for normality (Will-Shapiro’s test) and homoscedasticity (Levene’s test). Behavioral data were compared by ANOVAs, followed by Tukey’s HSD test, when appropriate.

Electrophysiological data were compared by paired or unpaired Student’s *t*-test.

All analyses were performed using Statistica 7.0 for Windows, and differences were considered significant at *p* ≤ 0.05.

## Results

### Behavioral effects of drugs acting on the endocannabinoid and dopaminergic systems

#### A/A Y-Maze

**Figure 2:**
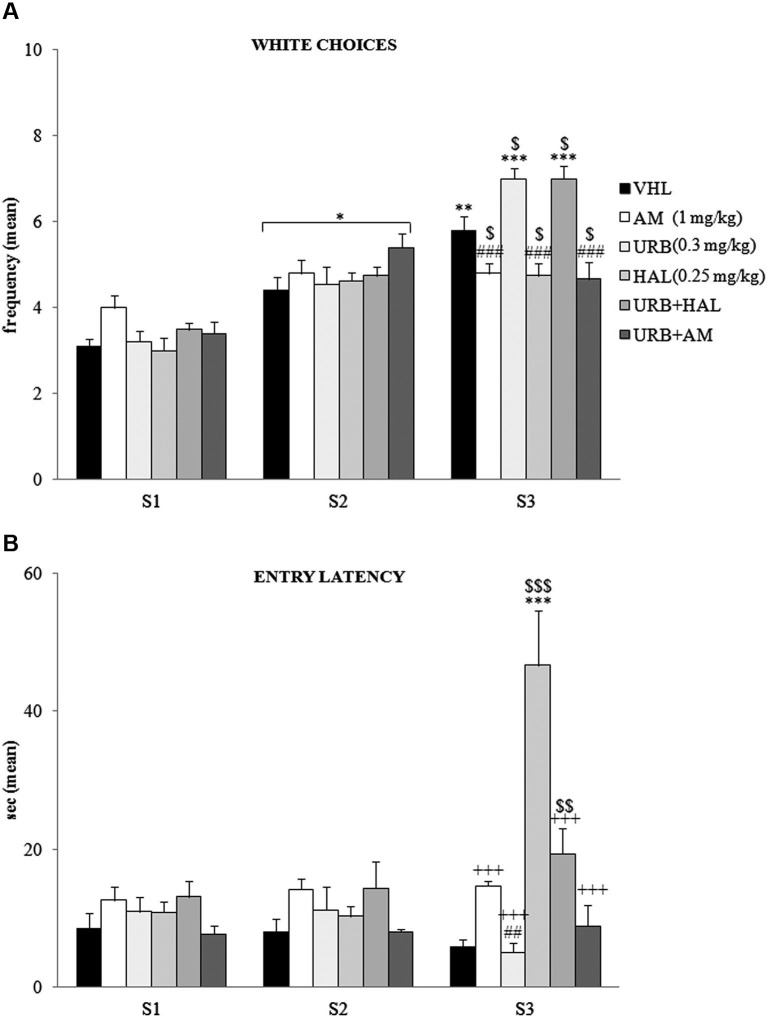
**Behavioral effects of A/A Y-Maze**. **(A)** Between S1 and S2, all animals displayed a significant (at least *p* = 0.02, *) increase in **white choices**. Between S2 and S3, the VHL (*p* = 0.006, **), URB (*p* = 0.0001, ***) and URB+HAL (*p* = 0.0001, ***) groups increased their white choices, while the AM, URB+AM and HAL groups did not. The significant *post hoc* comparisons of the intergroup differences in S3 were: URB (or URB+HAL) *vs.* AM or HAL or URB+AM: *p* = 0.0002, ###; VHL* vs.* all the other groups: at least*p* = 0.05, $. **(B)** Between S1 and S2, no significant differences in **entry latency** were found. Between S2 and S3, the HAL group had higher latency values (*p* < 0.0001, ***). In S3, the HAL group showed highest entry latency values (*vs.* URB, URB+HAL, AM, URB+AM groups at least *p* = 0.0001, +++; *vs.* VHL group *p* = 0.0001, $$$). The haloperidol powerful effect was contrasted but not prevented by URB597 and haloperidol coadministration, as the URB+HAL group had higher latency values than the URB (*p* = 0.002, ##) and VHL (*p* = 0.005, $$) groups.

The A/A Y-Maze required animals to choose between two conflicting drives, reaching a palatable reward in an aversive (white and lighted) arm or standard food in a reassuring (black and dark) arm. When white choice frequency was analyzed (Figure 2A) by two-way ANOVA (drug × session), there was significant effects of drug (*F*_(5,54)_ = 2.66; *p* = 0.03) and session (*F*_(2,108)_ = 147.83; *p* < 0.00001). Also, their interaction was significant (*F*_(10,108)_ = 10.22; *p* < 0.00001). *Post hoc* comparisons on the interaction revealed that the white arm in S1 was chosen least often by the animals of all groups. The frequency of white choices significantly (at least* p* = 0.02) increased in S2.

Between S2 and S3 the VHL group significantly (*p* = 0.006) increased their white choice. This response was expected, based on the anxiolytic and familiarization effects with the experimental apparatus with subsequent sessions. The URB group also significantly (*p* = 0.0001) chose the white arm more frequently, whereas the AM, URB+AM, and HAL groups did not. Thus, CB_1_ and DAergic antagonists impeded the increased choice of the white arm.

Notably, in URB+HAL group the coadministration of drugs significantly (*p* = 0.0001) increased the frequency of white choices to the same extent as in the URB group. *Post hoc* comparisons relative to the intergroup differences in S3 are reported in the Figure 2A.

With regard to entry latency (Figure 2B), by two-way ANOVA (drug × session), there were significant drug (*F*_(5,54)_ = 6.87; *p* = 0.00005) and session (*F*_(2,108)_ = 14.02; *p* = 0.000004) effects. Also, their interaction was significant (*F*_(10,108)_ = 15.57; *p* < 0.00001). By *post hoc* comparisons on interaction, there were no significant differences in latency values between S1 and S2.

In S3, the HAL group had the highest latency values compared with the other groups (all *p* = 0.0001)—when they stood still, they were in stable equilibrium with a broad-based support and when they were moving, they were slower to initiate (akinesia) and execute movements (bradykinesia). This significant motor slowdown was mitigated in part by the coadministration of URB597 and haloperidol. The URB+HAL group had significantly (*p* = 0.0001) lower latency values than the HAL group, higher values than the URB (*p* = 0.002) and VHL (*p* = 0.005) groups, and similar values as the AM and URB+AM groups. *Post hoc* comparisons are reported in the Figure 2B.

With regard to defecation boluses, two-way ANOVA (drug × session) indicated no significant drug (*F*_(5,54)_ = 2.45; *p* = 0.06) and session (*F*_(2,108)_ = 1.25; *p* = 0.29) effect. Also, their interaction was not significant (*F*_(10,108)_ = 1.46; *p* = 0.16).

#### OF

**Figure 3:**
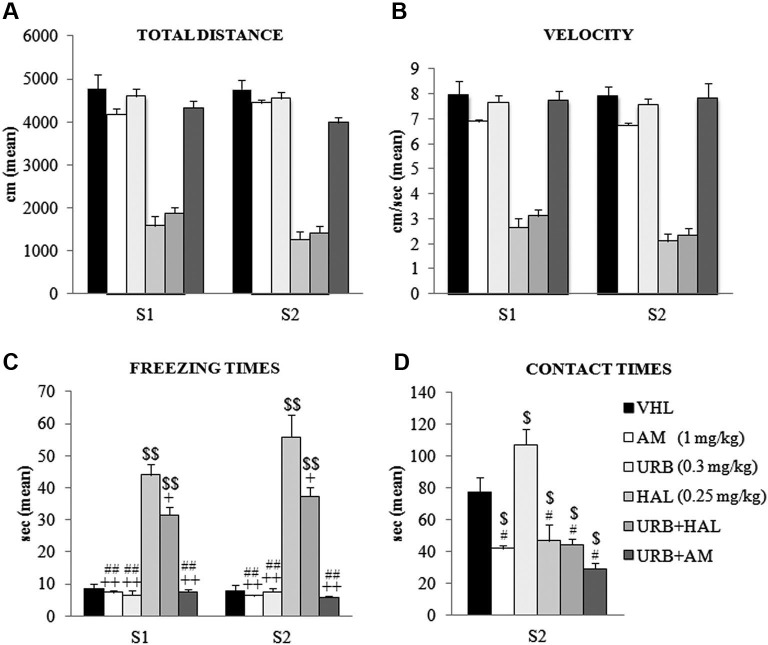
**Behavioral effects of OF task**. **(A)** Total distance, **(B)** velocity, **(C)** freezing times and **(D)** contact times in the absence (S1) and presence (S2) of the object. Note the haloperidol-dependent motor inhibition that was not contrasted (or only partially with regard to freezing times) by URB597 and haloperidol coadministration. In S1 and S2, the HAL group had the highest freezing times (*vs.* URB, URB+HAL, AM, URB+AM groups at least *p* = 0.01, +; *vs.* VHL group *p* = 0.001, $$). The URB+HAL group had freezing times higher than URB, AM, URB+AM (*p* = 0.001, ##) and VHL (*p* = 0.001, $$) groups. In **(D)**, the URB group displayed the highest contact times (*vs.* URB+HAL, URB+AM, HAL, AM groups at least *p* = 0.01, # *vs.* VHL group* p* = 0.05, $). All groups exhibited contact times significantly (at least *p* = 0.04, $) lower than the VHL group.

The animals were tested in an OF, in which the conflicting situation is represented by inserting an appealing new object in the anxiogenic central location of a wide arena. Two-way ANOVA (drug × session) on total distance traveled in the absence (S1) and presence (S2) of the object revealed a significant effect of drug (*F*_(5,54)_ = 108.52; *p* < 0.00001) but not session (*F*_(1,54)_ = 1.86; *p* = 0.17). Their interaction was not significant (*F*_(5,54)_ = 1.42; *p* = 0.23). *Post hoc* comparisons on the drug effect indicated that the haloperidol-dependent motor slowdown in the A/A Y-Maze was present also in the OF task. Notably, this effect was not relieved by the coadministration of URB597 and haloperidol–HAL and URB+HAL groups had similar distance values (*p* = 0.90). Both groups differed significantly from the remaining groups (all *p* = 0.0001). Conversely, the AM, URB, URB+AM, and VHL groups traveled similar distances (Figure 3A).

A similar pattern emerged with regard to mean velocity. Two-way ANOVA (drug × session) showed that drug had a significant effect (*F*_(5,54)_ = 81.16; *p* < 0.00001) but session did not (*F*_(1,54)_ = 3.19; *p* = 0.08); their interaction was not significant (*F*_(5,54)_ = 0.97; *p* = 0.44). *Post hoc* comparisons on the drug effect remarked the haperidol-dependent motor slowdown, which was not alleviated by URB597 plus haloperidol; the HAL and URB+HAL groups had similar velocity values (*p* = 0.95). Both groups differed significantly from the remaining groups (all *p* = 0.0001); the AM, URB, URB+AM, and VHL groups had similar velocities (Figure 3B).

Two-way ANOVA (drug × session) on *peripheral distance* revealed significant drug (*F*_(5,54)_ = 4.10; *p* = 0.003) and session (*F*_(1,54)_ = 152.80; *p* < 0.00001) effects. Also, their interaction was significant (*F*_(5,54)_ = 2.80; *p* = 0.02). *Post hoc* comparison on the interaction showed that all groups significantly (at least* p* = 0.05) reduced peripheral distance, from 70% in S1 to 40% of total distance in S2. This significant exploration of the central sectors in the arena during S2 was related to the presence of the new object and indicated that all groups had similar anxiety levels.

Two-way ANOVA (drug × session) on freezing times indicated that the effects of drug (*F*_(5,54)_ = 83.48; *p* < 0.00001), session (*F*_(1,54)_ = 3.79; *p* = 0.05), and their interaction were significant (*F*_(5,54)_ = 2.77; *p* = 0.02). *Post hoc* comparisons on interaction also showed the significant haloperidol-dependent motor inhibition. The HAL group had the highest freezing times in S1 (at least *p* = 0.01) and S2 (all *p* = 0.0001), an effect that was partially mitigated by the coadministration of URB597 and haloperidol. In S1 and S2, the URB+HAL group had significantly lower freezing times than the HAL group, although significantly (all *p* = 0.0001) higher than the other groups (Figure 3C).

For defecation boluses, two-way ANOVA (drug × session) revealed no significant drug (*F*_(5,54)_ = 2.03; *p* = 0.08) or session (*F*_(1,54)_ = 1.35; *p* = 0.31) effect. Their interaction was not significant (*F*_(5,54)_ = 1.96; *p* = 0.46).

One-way ANOVA on contact times revealed a significant drug effect (*F*_(5,54)_ = 15.62; *p* < 0.00001). *Post hoc* comparisons showed that the URB group had the highest contact times compared with all groups (at least* p* = 0.05). This effect was negated by the action of CB_1_ inverse agonist, AM251. The AM and URB+AM groups had similar contact times, which were significantly (at least* p* = 0.01) lower than in the VHL group. Also, the HAL and URB+HAL groups had similar contact times that were significantly (*p* = 0.04) lower than in the VHL group (Figure 3D). Thus, the decreased activity of CB_1_ receptors by AM251 (alone or with URB597) or of D_2_ receptors by haloperidol (alone or with URB597) inhibited the response to novelty.

### Electrophysiological effects of drugs acting on the endocannabinoid and dopaminergic systems

**Figure 4:**
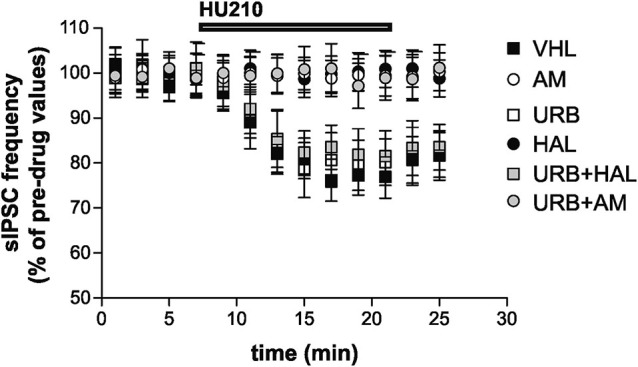
**Electrophysiological effects.** Stimulation of CB_1_ receptors with HU210 caused inhibition of striatal GABAergic sIPSCs in the VHL group. AM251 (alone or with URB597) and haloperidol treatments resulted in the suppression of HU210 effects. CB_1_ receptor sensitivity was rescued when haloperidol and URB597 were coadministered, although URB597 alone did not increase HU210 effects.

Stimulation of CB_1_ receptors by HU210 significantly inhibited striatal GABAergic sIPSCs in the VHL group (*n* = 9, *p* < 0.01). *In vivo* treatment with AM251 failed to alter *per se* sIPSC frequency (*n* = 12, *p* > 0.05 compared with the VHL group before HU210) (data not shown) but suppressed the effects of HU210 (*n* = 10, *p* > 0.05). This effect was also present in the URB+AM group (*n* = 11, *p* > 0.05). Haloperidol blocked the effects of HU210 on striatal GABAergic synapses (*n* = 6, *p* > 0.05). Notably, CB_1_ receptor sensitivity was rescued by coadministration of haloperidol and URB597 (*n* = 8, *p* < 0.05), although URB597 alone did not increase *per se* the HU210 effects on sIPSCs (*n* = 6, *p* < 0.01 compared with the pre-HU210 value and *p* n.s. compared with effect of HU210 in the VHL group) (Figure 4).

## Discussion

In the behavioral tests that we administered, there was a conflict between positive and negative drives; thus, approach and avoidance behaviors were evoked simultaneously. Typically, when these trends have similar strength, the subject remains suspended or, at best, tends to gravitate toward the heavier pole of the conflicting situation. When the subject is re-exposed to the same conflicting situation, the anxiogenic components tend to fade, and the more appealing pole of the conflicting situation is reached more easily. These reasons are why the control animals (VHL group) moved slightly toward the appetitive (palatable food or new object) pole and increased the responses to reward when re-exposed to the task in the A/A Y-Maze.

The main finding of this study was that CB_1_-mediated processes and their interaction with DAergic transmission modulated the salience of reward. In S3 of the A/A Y-Maze and in the OF task, animals that were treated with URB597, a drug that potentiates endocannabinoid activity through anandamide increase, had a robust approach behavior toward palatable food and the new object. Notably, AM251, a CB_1_ inverse agonist, alone and in combination with URB597, blocked the approach behavior, demonstrating that the effect of URB597 on such behavior is mediated by CB_1_ receptors. Recently, the orphan G-Protein coupled Receptor, GPR55, has been presented as a candidate of cannabinoid receptor subtypes (Ryberg et al., 2007; Lauckner et al., 2008). The GPR55 recognizes cannabinoids, but it differs from CB_1_ receptor. Some cannabinoids have high affinity for the GPR55 receptor and in low doses can play as an agonist for this receptor. Also the AM251 behaves as an agonist with high affinity for GPR55 receptor (Ryberg et al., 2007). Therefore, in the present study the blockade of the approach behavior observed in the presence of AM251 administration could be related also to the activity of the GPR55 receptors.

CB_1_ are expressed in several reward-related brain areas, such as the substantia nigra, ventral tegmental area, dorsal and ventral striatum, prefrontal cortex, and corticolimbic structures that receive collateral DAergic innervations (Marsicano and Lutz, 1999; Hermann et al., 2002). Specifically, in the dorsal striatum, CB_1_ expression is significant in medium spiny neurons that receive DAergic inputs and express D_1_ and D_2_ receptors (Monory et al., 2007). Striatal DA efflux rises when anandamide levels are upregulated by URB597 (Solinas et al., 2006). However, in striatal slice preparations, CB_1_ receptor activation has no effect on DA release (Köfalvi et al., 2005), suggesting that CB_1_ regulation of DA release involves a multisynaptic mechanism (Riegel and Lupica, 2004; Marinelli et al., 2007).

The administration of haloperidol (D2 antagonist) blocked approach behavior, like AM251 did. However, whereas AM251 did not influence motor function, haloperidol impaired locomotor activity. Consistent with this observation, much evidence indicates that this neuroleptic drug causes dose-dependent akinesia and muscle rigidity (De Ryck et al., 1980; Lorenc-Koci et al., 1996; Ozer et al., 1997).

Coadministration of URB597 and haloperidol counteracted the effects of haloperidol on approach behavior and motor activity. Notably, the effect of ECS potentiation, combined with D_2_ receptor blockade, developed only when the reward was represented by palatable food. Such a facilitatory effect on food reinforcement appeared to be due to the higher salience of palatable food, based on the hedonic properties of its palatability, compared with the lower salience of the object, regardless of its novelty. The regulation of DAegic processes by the striatal ECS is central to the hedonic aspects of food-seeking (Duarte et al., 2004), food novelty (Bassareo et al., 2002), and need state with regard to hunger (De Luca et al., 2012). CB_1_ antagonists decrease and CB_1_ agonists increase DA release that is induced by rewarding stimuli (Cohen et al., 2002; Fadda et al., 2006; Solinas et al., 2006).

In this study, the combination of URB597 and haloperidol affected motor activity. Although slackened, URB+HAL-treated animals had lower entry latencies in the A/A Y-Maze but remained inhibited in the OF task, as evidenced by their total distance and velocity values. Such dissociation between behaviors in the tasks suggests that the reduction in entry latency values in the A/A Y-Maze was a food-dependent effect. The motor slowdown that was evoked by the D_2_ antagonist was mitigated in the URB+HAL group only when stimulus salience was high, as for palatable food. Likely, the haloperidol increases the amount of glutamate that is released from corticostriatal neurons by counteracting the DA-dependent inhibition of this release, which in turn stimulates striatopallidal output neurons and renders the animal akinetic with muscle rigidity (Yoshida et al., 1994).

It has been suggested that NMDA antagonists alleviate motor slowdown by suppressing the excessive cortical stimulation of the striatopallidal pathway at the level of the striatum (Kaur et al., 1997). Thus, in the URB+HAL group, the partial motor recovery might be linked to the ability of URB597 to potentiate endocannabinoid tone, which in turn activates CB_1_ receptors that presynaptically inhibit striatal glutamatergic neurotransmission. This proposal is supported by Giuffrida et al. (1999), who demonstrated that motor activity increases anandamide dorsostriatal release after administration of a D_2_ agonist, a response that is prevented by a D_2_ antagonist. Further, D_1_ agonists did not change the basal outflow of anandamide, underscoring the differences between D_1_ and D_2_ agonists with respect to anandamide release.

Notably, blockade of CB_1_ activity has opposite effects on psychostimulant-induced hyperactivity (Poncelet et al., 1999). Conversely, inhibitors of the anandamide reuptake or of FAAH attenuate the hyperactivity in hyperdopaminergic mice (Tzavara et al., 2006). Hyperactivity also decreases in response to d-amphetamine treatment in CB_1_ knockout mice (Tzavara et al., 2009).

Because the behavioral tests of this study integrated an approach-avoidance conflict (reward searching or exploratory drive against brightly lit or open space), the inevitable anxiogenic component that is linked to the conflict must be considered. At least two factors can influence anxiety-like behavior in the A/A Y-Maze and OF tasks: social isolation of the single specimen, resulting from physical separation from cage mates when performing the test, and the aversive feature that is created by the brightly lit, unprotected, novel environment. Thus, both tests can be used to screen for anxiety-related behaviors and analyze the impact of drugs on them.

Drugs that target the ECS elicit anxiolytic or anxiogenic actions. Specifically, in various anxiety paradigms, URB597 has anxiolytic effects (Patel and Hillard, 2006; Moreira et al., 2008; Rubino et al., 2008; Scherma et al., 2008), depending largely on the stress conditions of the experimental protocols, provided that the conditions do not exceed the ECS buffering function (Naidu et al., 2007; Haller et al., 2009). These findings indicate tonic modulation of aversive responses, based on the approach-avoidance conflict. Further, CB_1_ receptor agonists induce biphasic effects, wherein lower doses are anxiolytic and higher doses are anxiogenic (Viveros et al., 2005). AM251 has an anxiogenic effect when injected at high (3.0 mg/kg) doses and reverses URB597-induced anxiolytic and panicolytic effects (Gobira et al., 2013).

In this study, all groups that were treated with drugs that act on the ECS and DAergic system had similar anxiety levels as the VHL group, based on analysis of primarily anxiety-related parameters—e.g., A/A Y-Maze and OF defecation boluses and OF peripheral distance (thigmotaxis). With regard to OF freezing times, whereas animals that were given ECS-targeted drugs had similar values, the HAL and URB+HAL groups had significantly higher freezing times. However, this increase can not be considered an index of merely enhanced anxiety, because it was heavily influenced by the confounding factor haloperidol-dependent motor slowdown. These findings are consistent with the hypothesis that a fear/anxiety state does not underlie haloperidol-induced catalepsy (Colombo et al., 2013). Similarly, there is no evidence of a relationship between catalepsy and fear/anxiety state in congenic mouse strains (Kondaurova et al., 2011).

Our electrophysiological results complement our behavioral findings. In the VHL and URB groups, stimulation with HU210 (CB_1_ agonist) inhibited GABAergic dorsostriatal neurotransmission, consistent with previous reports (De Chiara et al., 2010b; Laricchiuta et al., 2012b). In rodents, manipulations with strong reinforcing properties, such as cocaine-induced conditioned place preference, spontaneous running wheel activity, and sucrose consumption, are associated with hypersensitivity of dorsostriatal GABAergic synapses to CB_1_ stimulation (Centonze et al., 2007a, b; De Chiara et al., 2010b). In a recent study (Laricchiuta et al., 2012b), we reported that enhanced or reduced CB_1_-mediated control over GABAergic dorsostriatal neurotransmission was associated with spontaneous approach or avoidance behavior toward or away from palatable food, respectively. Further, CB_1_ activation on GABAergic or glutamatergic neurons has opposite effects on exploratory activity (Häring et al., 2011).

In this study, the administration of the CB_1_ inverse agonist AM251 (alone or with URB597) or the D_2_ antagonist haloperidol suppressed the effects of HU210 on GABAergic dorsostriatal transmission. These findings are consistent with the observation that D_2_ stimulation activates the dorsostriatal ECS, influencing GABAergic synapses (Centonze et al., 2004, 2007a, b). Notably, in our study, CB_1_ receptor sensitivity to HU210 was rescued when URB597 and haloperidol were coadministered.

Overall, our behavioral and electrophysiological results demonstrated that by increasing anandamide levels, endocannabinoid potentiation magnified the search for reward and, in parallel, inhibited dorsostriatal GABAergic neurotransmission. Blockade of CB_1_ or D_2_ receptors inhibited reward-related responses and prevented the inhibition of dorsostriatal GABAergic neurotransmission. Notably, the reward-related response was restored when the blockade of DAergic activity was combined with ECS potentiation. This effect occurred only if the reward was palatable food. Accordingly, the coadministration of URB597 and haloperidol restored the dorsostriatal responses to stimulation with HU210.

Although the use of single doses of drugs might be a limitation in interpreting behavioral and electrophysiological effects we found, it has to be underlined that our results are fully consistent with the hypothesis that endocannabinoids control the reward-related processes and are also implicated in reward-related disorders. Endocannabinoid and DAergic transmission might interact functionally to modulate salient information processing. Abnormalities in the neural mechanisms that govern reward-related processes might underlie the aberrant emotional processing in such disorders as schizophrenia and addiction (Ziauddeen and Murray, 2010; Gardner, 2011). The increase in anandamide in schizophrenic patients might constitute a compensatory response to counteract primary DAergic dysfunction (Giuffrida et al., 2004), advancing the therapeutic potential of the ECS in DA-related disorders. Thus, new insight into ECS activity in reward-related DAergic circuitry should guide the development of pharmacological treatments for eating, drug abuse, and psychiatric disorders.

## Author contributions

Daniela Laricchiuta and Diego Centonze designed research; Daniela Laricchiuta, Alessandra Musella and Silvia Rossi performed research; all authors analyzed, discussed and interpreted the data; Daniela Laricchiuta and Diego Centonze wrote the paper and revisited it critically for important intellectual content; all authors approved the final version of the paper and they agreed to be accountable for all aspects of the work.

## Conflict of interest statement

The authors declare that the research was conducted in the absence of any commercial or financial relationships that could be construed as a potential conflict of interest.
